# Identifying Peripheral Neuropathy in Colour Fundus Photographs Based on Deep Learning

**DOI:** 10.3390/diagnostics11111943

**Published:** 2021-10-20

**Authors:** Diego R. Cervera, Luke Smith, Luis Diaz-Santana, Meenakshi Kumar, Rajiv Raman, Sobha Sivaprasad

**Affiliations:** 1 Cambridge Consultants, Science Park, Milton Road, Cambridge CB4 0DW, UK; diego.cervera@cambridgeconsultants.com (D.R.C.); luke.d.smith@cambridgeconsultants.com (L.S.); luis.diaz-santana@cambridgeconsultants.com (L.D.-S.); 2 Shri Bhagwan Mahavir Department of Vitreoretinal Services, Sankara Nethralaya, No. 41 (Old 18), College Road, Chennai 600 006, India; meenueso@gmail.com; 3 NIHR Moorfields Biomedical Research Centre, 162 City Road, London EC1V 2PD, UK; senswathi@aol.com

**Keywords:** diabetes, deep learning, diabetic neuropathy, diabetic retinopathy

## Abstract

The aim of this study was to develop and validate a deep learning-based system to detect peripheral neuropathy (DN) from retinal colour images in people with diabetes. Retinal images from 1561 people with diabetes were used to predictDN diagnosed on vibration perception threshold. A total of 189 had diabetic retinopathy (DR), 276 had DN, and 43 had both DR and DN. 90% of the images were used for training and validation and 10% for testing. Deep neural networks, including Squeezenet, Inception, and Densenet were utilized, and the architectures were tested with and without pre-trained weights. Random transform of images was used during training. The algorithm was trained and tested using three sets of data: all retinal images, images without DR and images with DR. Area under the ROC curve (AUC) was used to evaluate performance. The AUC to predict DN on the whole cohort was 0.8013 (±0.0257) on the validation set and 0.7097 (±0.0031) on the test set. The AUC increased to 0.8673 (±0.0088) in the presence of DR. The retinal images can be used to identify individuals with DN and provides an opportunity to educate patients about their DN status when they attend DR screening.

## 1. Introduction

Diabetes mellitus (DM) is a significant public health problem globally. According to the International Diabetic Federation’s (IDF), about 425 million adults in 2017 were living with diabetes globally; by 2045, this number is projected to rise to 629 million [1]. Additionally, over the past decade, the prevalence of diabetes has risen faster in low and middle-income countries than in high-income ones [2].

Peripheral neuropathy is a common complication of DM that can result in significant morbidity and disability due to foot ulceration and amputation. In countries such as India, the lifetime risk of a person with diabetes having a foot ulcer has been reported to be as high as 25%, with approximately 100,000 people requiring lower limb amputation annually [3]. An estimated 46.1% of diabetic foot ulcers are neuropathic, while the rest may be ischaemic or a combination of ischaemic and neuropathic [4]. Screening for high-risk diabetic neuropathy (DN) and early management reduces the risk of ulcers by 60% and amputations by 85% [5]. Several methods are used to detect peripheral neuropathy ranging from simple tests such as a tuning fork or monofilament test to more accurate devices such as the biothesiometer, handheld Doppler, or the pedometer. The vibration perception threshold (VPT) measured by a biothesiometer is an accurate quantitative indicator of clinical neuropathy [6]. However, these kits are not always widely used in low-resource settings. In addition, it is important to reiterate the need for consulting for their DN while attending DR screening, so that people with diabetes are treated for all their complications early.

Opportunistic screening for diabetic retinopathy (DR) using colour fundus photographs (retinal images) is widely practised. However, as reported from previous studies, not all people with DR have DN and vice versa [7]. Therefore, we hypothesized that if DN could also be identified from retinal images independent of DR, it will facilitate dual screening of DN and DR using retinal images. Using visual inspection alone of fundus images is currently not possible.

Machine learning has been used for the automated classification of DR from retinal images [8]. Many deep learning-based algorithms have shown comparable accuracies to that of human experts [9]. Deep learning-based algorithms on retinal images have also been developed and validated to predict multiple cardiovascular risk factors, including age, gender, and systolic blood pressure [10]. Recently, the estimated glomerular filtration rate (eGFR) was accurately predicted from retinal images using deep-learning methods [11]. In this study, we aimed to evaluate whether a deep learning algorithm could be trained to predict the diagnosis of DN on retinal images in people with diabetes. We hypothesized that the deep learning algorithm could detect features or patterns that are not evident on standard clinical reviews of retinal images.

## 2. Materials and Methods

Institutional review board approval was obtained for retrospective analysis of prospectively collected data from a population-based study in South India, Sankara Nethralaya Diabetic Retinopathy Epidemiology and molecular genetics study (SNDREAMS). Written informed consent was not required as this study was an analysis of previously collected anonymized data and retinal images.

In SNDREAMS, 6000 people aged ≥40 years were recruited from urban Chennai using multistage systematic random sampling between 2003–2006 to assess the prevalence and incidence of DR and its risk factors. Detailed protocols for obtaining the measurements from participants have been described elsewhere [12]. A total of 1561 (26%) people were diagnosed with diabetes from this study based on two readings of fasting blood sugar of >110 mg/dL, or a history of known diabetes on medications. The diagnostic of each patient was for a single point in time, and therefore unique.

### 2.1. Assessment of Diabetic Neuropathy (DN)

In the SNDREAMS study, DN assessment was done on all participants with diabetes by measuring VPT using a biothesiometer (Kodys, Chennai, India). The VPT was measured by a single observer by placing the biothesiometer probe perpendicular to the distal plantar surface of the great toe of both legs. The VPT was measured at a voltage level when the patient felt the first vibration sensation. The mean VPT measure of three readings of both legs was considered for the analysis. The diagnosis of DN was made if the VPT value was ≥20 V6.

### 2.2. Assessment of Diabetic Retinopathy (DR)

All participants with diabetes underwent retinal photographs using Carl Zeiss fundus camera (Visucamlite, Jena, Germany). DR was classified according to the modified Klein classification (Modified Early Treatment DR Study scales [13]) by two independent observers in a masked fashion with a high agreement (k = 0.83).

### 2.3. Data Analysis

#### 2.3.1. Dataset Splits

For this study, 23,784 retinal images from 1561 participants with diabetes were used. Of these, the images of 1401 participants (90% of the total) were used for model training and validation, and the images of the remaining 160 participants (10%) were saved for testing (refer to the Training section for more details). For both training and testing, the corresponding set of images was used as a whole, but also two subsets were used: a set containing only patients with DR, and a set containing only patients without DR (refer to Table 1 for definitions).

#### 2.3.2. Dataset Processing

The data available contained a single diagnostic per patient (for DR and DN) and an average of 16 images per patient (of both eyes). Each of the images of the patient was then labelled with the patient diagnostic (presence/absence of DN/DR). When DR was only present in one of the eyes of the patient, or when any of the data entries were not valid, the patient and their images were removed from the dataset. The final numbers of patients and images used for training/testing are shown in Table 1 (Insert Table 1).

No image pre-processing or filtering was used as a means of cleaning the dataset; the images were used as provided (including noisy images such as blurred, with bright corneal reflections and external images of the eye). The images were re-sized using bilinear interpolation to 720 × 576 px to reduce the amount of memory required. Data augmentation was used during training, using a series of random transforms (horizontal and vertical flip, rotations (±90°), as well as random modifications of the brightness (±0.2), contrast (±0.2), saturation (±0.2), and hue (±0.2) (When using data augmentation, the original image is randomly modified every time it is fed to the model. That way, at every epoch, the model will see variations of the original images that it has not seen before. One epoch is when all the images of the training set have been used exactly once to update the internal parameters of the model).

### 2.4. Training

The training on DN was carried out by performing a grid search for exploring different architectures (algorithm configurations) and combinations of hyper-parameters. Grid search allows for a thorough exploration of the architecture/hyper-parameter space and it generates all possible combinations of hyper-parameters, each of these combinations defining ‘a model’. Thus, a model has a defined architecture, and a fixed set of hyper-parameters (see Table 2 for a detail of the hyper-parameters in each model). The architecture is considered a hyper-parameter in this paper. All hyper-parameters chosen remained fixed throughout training and testing.

The architectures we tested were Deep Neural Networks: Inception v3 [14], Squeezenet v1.0 [15] and Densenet 121 [16]. Each of them was tested with and without pre-trained weights (trained on the ImageNet dataset). The other hyper-parameters explored were the optimiser Adam [17] or SGD [18], its learning rate (values in the range 10 × 10^−6^–10 × 10^−2^) and momentum (when applicable) and the dropout rate (0.3–0.7).

A final hyper-parameter was defined to investigate two different ways of dealing with the class imbalance, that is, to deal with the fact that the distribution of patients with DN and DR are much smaller than the size of the sample. The two options explored to address class imbalance were weighted loss and weighted sampling. The weighted loss applies a re-scaling weight to the loss associated, with each class inversely proportional to the number of occurrences of that class in the dataset; in that way, the misclassification of the least frequent class had a bigger impact per sample on the loss value. Using weighted sampling, each sample of under-represented classes was reused more often (and augmented, to avoid overfitting) to give the appearance of balanced occurrences of each class.

The training process consisted of 5-fold cross-validation: the training data is divided into five splits of similar size and similar distribution (with no patient overlap between splits), and there are five rounds of training and validation wherein each round four splits of the data are used for training, and one for validation. The validation split is different for each round, and it is used during the training phase to prevent over-fitting to the training set: at regular intervals throughout training, the performance of the model is evaluated against the validation split. When the performance of the model on the validation split starts to deteriorate, training is stopped (early stopping).

We chose the 5-fold cross-validation approach to ensure that the observed performance of a model was representative of the sample and not due to a lucky split (where the samples in the validation set are easier to predict on average than what it would be in the real distribution). All the splits were stratified so that the distribution of the patients with DN/DR was approximately the same as the true distribution in the dataset, and the performance on each of the folds was the average performance of the model.

Given the large amount of hyper-parameter combinations (models), running the 5-fold cross-validation training for all of them would have been very time/resource consuming. Hence, the training was carried out in two phases (See Table 3):

First phase: all models were trained on a single fold and evaluated using the validation split of that fold (the test set was not used in this phase). The grid search used in this phase of training can be seen as a random walk approach for finding the best model (and hence, the best set of hyper-parameters). The models represent points in the hyper-parameter space, and their performance (measured using the area under the rate receiver operating characteristic curve (AUC)) defines the topography of the space.

Second phase: the model with the best performance from the First Phase (higher AUC) was then trained using the 5-fold cross-validation method. The final performance of the model was evaluated using the test set.

**Figure 1:**
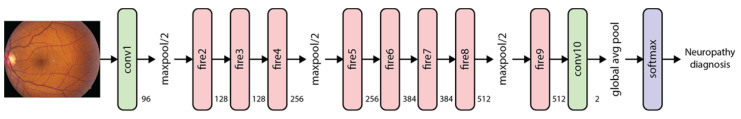
Squeezenet architecture.

After the exploration of architectures and hyper-parameters, the selected architecture was the Squeezenet (Figure 1).

This is a Deep Neural Network that uses a series of “Fire” modules alternated with max-pool layers. Each “Fire” module consists of a squeeze convolution layer (1 × 1 filters), combined with an expand layer (a combination of 1 × 1 and 3 × 3 convolutions), using ReLUs as activation functions [19]. The final block is a convolutional layer followed by a global average pool layer. The selected optimiser was Adam [17] (with a learning rate of 2.7 × 10^−6^); the dropout rate was set to 0.70, and the input consisted of batches of images of size 8. The training stopped after 100 epochs when early stopping was triggered to prevent overfitting.

All training was run in a local cluster, using Docker containers running Ubuntu 16.04, with PyTorch 1.0.1. Each model/fold was run in a single NVIDIA GPU, with a minimum of 12 GB RAM, using CUDA 9.0 and CUDNN 7.

#### 2.4.1. Algorithm Evaluation

The best model chosen from the first phase was trained on the second phase using 5-fold cross-validation in each of the subsets mentioned above (trDR+, trDR−, trA). Note the distinction between a model, and a trained model: a model is defined by a fixed set of hyper-parameters; a trained model has, in addition, a set of weights that are learnt during training. All the models trained in the second phase share the same set of hyper-parameters, but their weights are unique. For readability, trained models will be referred to as models in this section.

We refer to the model trained on a specific subset by the name of the subset: e.g., the model that was trained on the subset trDR+ (only on images of patients with DR) will be referred to as trDR+.

Once trained, each of the three models (trDR+, trDR−, and trA) was tested on the three test sets (tsDR+, tsDR−, and tsA).

The reported performance is the AUC of a true/false positive rate receiver operating characteristic (ROC) curve of deep learning models and plotted with a 95% confidence interval (CI). No bootstrapping was used, and images were used just once per test. The true/false positive rate (TPR/FPR) is measured across images rather than patients. The results are plotted as a function of TPR-FPR, where TPR is the Sensitivity, and FPR is 1-Specificity20. For each model, the plots contain five different ROC curves: they represent the performance of the model trained on each fold of the 5-fold cross-validation subsets.

#### 2.4.2. Demographic Analysis

Statistical analysis was performed using SPSS software (IBM, SPSS 20). The comparison on demographics was done using Kruskal Wallis one-way analysis of variance (ANOVA). For nominal data, chi-square test was done. Statistical significance level was set at 0.05.

## 3. Results

A total of 189 (12.10%) patients had only DR, 276 (17.68%) had only DN, and 43 (2.75%) patients had both DR and DN. Table 4 shows the demographic details of the groups of patients There was a significant difference in between the groups in Duration of Diabetes and HbA1c, BMI, and cholesterol levels. The longest diabetes duration was observed in DN group while the highest HbA1C was observed in the retinopathy only group. Lowest HbA1c and serum cholesterol was observed in the “No retinopathy no neuropathy” group.

### Model Performance

**Figure 2:**
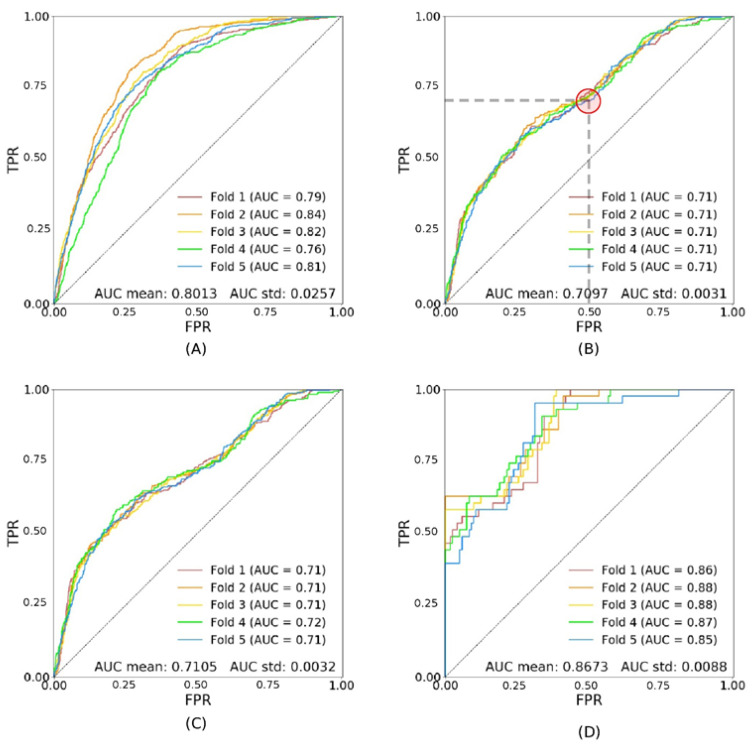
Shows the performance of the model trA (trained on all patients). Image (**A**) shows the ROC curves for the validation set (val); (**B**) shows the ROC curves for the test set with all patients (tsA), the red circle is a representative point on the curve, with 70% TPR and 50% FPR (see text for further details); (**C**) shows the ROC curves for the test set with patients without DR only (tsDR−); (**D**) shows the ROC curves for the test set with patients with DR only (tsDR+).

The ROC curves of the Squeezenet v1.0 network trained on 90% of the data and tested on the remaining 10% are presented. Figure 2 illustrates the performance of the model trA.

The AUC for the validation set is 0.8013 ± 0.0257 (Figure 2A), and 0.7097 ± 0.0031 in the test set (Figure 2B). A representative point on the ROC curve achieves a 70% TPR with 50% FPR (red circle on Figure 2B). When testing in the subsets tsDR− and tsDR+, the AUC is only significantly higher (0.8673 ± 0.0088, *p* < 0.0001) in the subset tsDR+ (Figure 2C,D).

**Figure 3:**
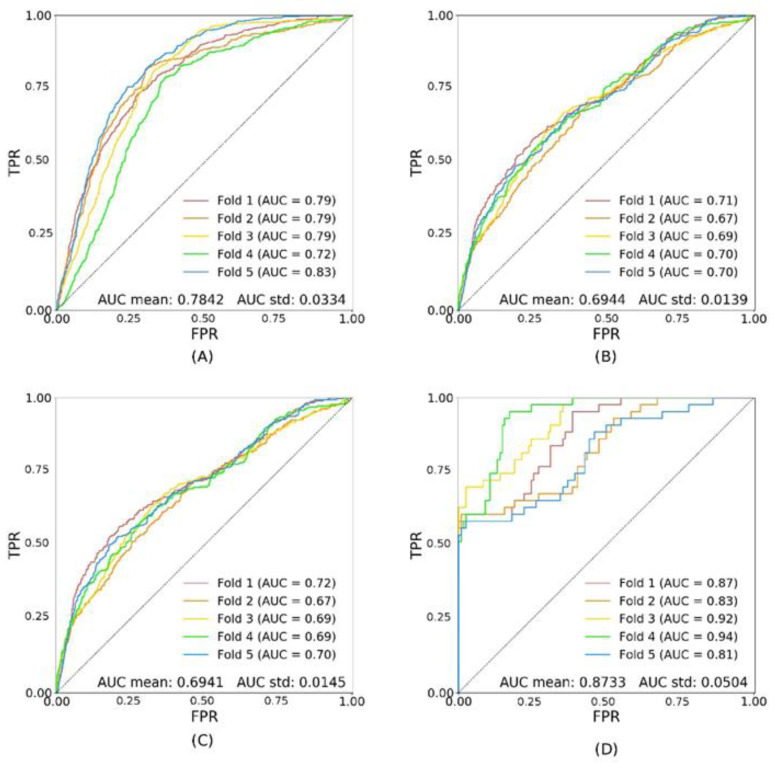
Shows the performance of the model trDR− (trained on patients without DR). Image (**A**) shows the ROC curves for the validation set (val); (**B**) shows the ROC curves for the test set with all patients (tsA); (**C**) shows the ROC curves for the test set with patients without DR only (tsDR−); (**D**) shows the ROC curves for the test set with patients with DR only (tsDR+).

**Figure 4:**
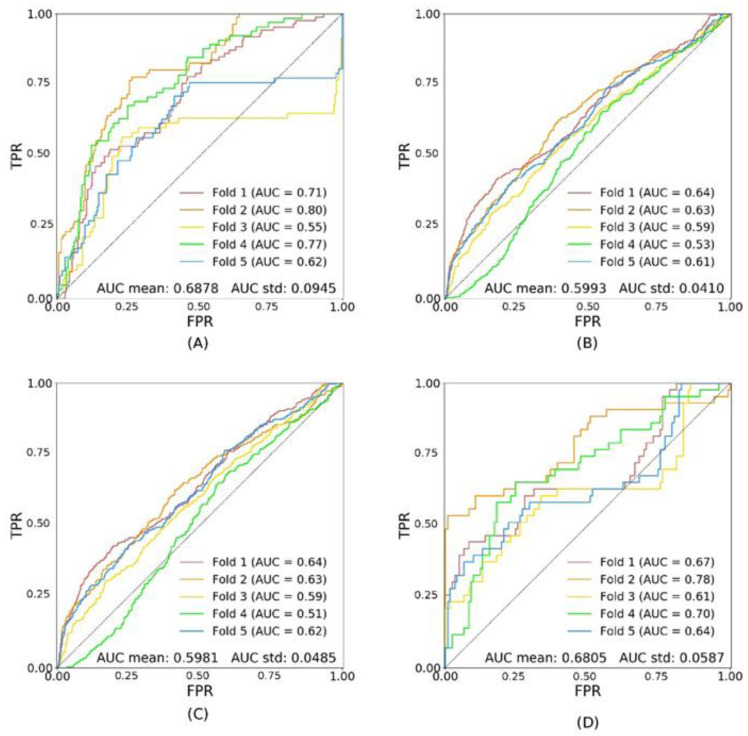
Shows the performance of the model trDR+ (trained on patients with DR). Image (**A**) shows the ROC curves for the validation set (val); (**B**) shows the ROC curves for the test set with all patients (tsA); (**C**) shows the ROC curves for the test set with patients without DR only (tsDR−); (**D**) shows the ROC curves for the test set with patients with DR only (tsDR+).

Figure 3 and Figure 4 show the results of inference generated by models trained on the subsets trDR− and trDR+, respectively. The performance of the model trained on trDR− is not significantly different from the performance of the model trained on trA. It achieves 0.6944 ± 0.0139 on tsA (Figure 3B). On the contrary, when trained on trDR+, the performance of the model is significantly lower (AUC: 0.5993 ± 0.0410 on tsA) (Figure 4B). Note that trA and trDR− have a similar number of patients in the training dataset (1081 and 988 individuals), whereas trDR+ has an order of magnitude fewer patients (93). This adds noise to the training and the predictions, as can be observed in the higher values of the standard deviations.

In all three experiments (trA, trDR−, and trDR+), the results obtained when testing on tsDR+ are significantly higher than those obtained when testing on tsA or tsDR− (*p* < 0.0001). The AUC scores for the models trained on the three different subsets of the dataset (trA, trDR−, and trDR+), tested on the test subsets (tsA, tsDR−, and tsDR+), are shown in Table 5.

## 4. Discussion

Our results indicate that the application of deep learning to retinal fundus images alone can be used to identify DN. We demonstrated for the first time that using retinal images from people with diabetes can be used to identify individuals with DN. Moreover, the predictive power of the model, i.e., its ability to predict DN, increases when DR is present. While the overall accuracy of this method likely requires refinement for clinical usage, this study reveals that screening DN using retinal images in people with diabetes shows promise.

The biothesiometer mainly tests the function of large nerve fibres and abnormal VPT is associated with significant nerve damage as in gangrene and foot ulceration [6]. This study shows that retinal images may be used as an alternative to VPT to evaluate peripheral large nerve fibres, especially in community-based DR screening programmes. Future work could evaluate if fundus images contain information from earlier stages of DN.

Neriyanuri et al. [20] showed that subjects with DN but no DR showed an increase in the foveal thickness, reduced RNFL thickness, and a significant reduction in visual functions, including visual acuity, contrast sensitivity, mean retinal sensitivity, and colour vision when compared with those without DN. Thus, there are functional changes in the retina in DN. So, there may be structural clues in the retinal image that may be picked up by the deep learning algorithm. As the use of OCT becomes more prevalent in primary care, more powerful screening options may also become available.

In our study, the predictive power of the algorithm increased with the presence of DR. Srinivasan et al. [21] in their longitudinal study had reported greater age, lower socioeconomic score, higher body mass index, and the presence of DR are risk factors for incident neuropathy, when adjusted for several potential confounding factors. Those with moderate and severe non-proliferative DR showed a significantly greater risk of developing DN than those with mild or no DR. This may be the probable reason for better performance of the algorithm in presence of DR.

We have provided evidence that deep learning may uncover additional signals in retinal images that will allow detection of DN. Despite the promising results, our study has several limitations. First of all, the type of Diabetes in this study was type II. To group the type of diabetes, a c-peptide test is usually done. However, this test was not used in this study and the grouping was done based on diabetic onset elicited from patient history and use of oral hyperglycemics. Literature also suggests that alcohol intake and vitamin deficiencies can lead to peripheral neuropathy, which unfortunately could not be explored in detail in this study due to its retrospective nature. Our study only used images with a 45º field of view, and future work could examine the generalizability of these findings to images with larger fields of view. In addition, the overall size of the dataset is relatively small for deep learning. The improved performance of the algorithm in patients with DR needs to be validated in a larger sample. Although we could demonstrate that the algorithm could predict DN with deep learning system by learning the disease, we are unaware of how the system detects the neuropathy from the fundus image, which is not visible to human eyes. It would be important to identify the heat maps which the algorithm used to do this prediction, which was out of scope in this study. The images were of lesser resolution than what is available today. Additionally, since the algorithm was trained with a noisy data set, it could be more realistic of what the real-world data may look like. In the future—in a clinical implementation—a cleaner data set could be used for training and a pre-cleaning algorithm could be used once deployed, likely improving the predictive power of the methodology described in this paper.

The results from our study demonstrate that the information required to predict the presence of NP was contained in these images, even at the lower resolution captures of the time. We would certainly expect that higher resolution images may contain more information and possibly better predictive power, and pave the way to the future. Our work also suggests avenues of future research into the source of these associations, and whether they can be used to better understand and prevent diabetic neuropathy.

## Figures and Tables

**Table 1:** Definition of the datasets used for training and testing.

Phase	Name	Description	Patients *	Images *
Training	trA	all images (patients both with and without DR)	1081	17,028
	trDR+	only images of patients with DR	93	1503
	trDR−	only images of patients without DR	988	15,525
Testing	tsA	all images (patients both with and without DR)	121	1892
	tsDR+	only images of patients with DR	11	165
	tsDR−	only images of patients without DR	110	1727
{trA} ∩ {tsA} = {Ø}; {trA} ∪ {tsA} = {A} {trDR+} ∩ {trDR−} = {Ø}; {trDR+} ∪ {trDR−} = {trA} {tsDR+} ∩ {tsDR−} = {Ø}; {tsDR+} ∪ {tsDR−} = {tsA}				

* The number of patients and images shown are the result of the dataset processing. Refer to Dataset processing section.

**Table 2:** Hyper-parameters explored in the grid search.

Hyper-Parameter	Values	Number of Values
Architecture	{Inception, Squeezenet, Densenet}	3
Optimiser	{SGD, Adam}	2
Learning rate	[10 × 10^−6^, 10 × 10^−2^]	10
Momentum	{0.95, 0.99}	2
Dropout	{0.3, 0.5, 0.7}	3
Class rebalancing	{weighted loss, weighted sampling}	2
**Total number of combinations/models**		**720**

**Table 3:** Training and evaluation process.

	Models	Trained on:	Performance (AUC) on:
**Phase 1**	All models (see Table 2)	Training set, split 1	Validation set, split 1
**Phase 2**	Best model from Phase 1	Training set, all splits	Test set (average AUC)

**Table 4:** Demographics and clinical characteristics of the patients.

	Disease State	No Diabetic Retinopathy/Neuropathy	Diabetic Retinopathy	Diabetic Neuropathy	Combined	
Variables		*n* = 1101	*n* = 189	*n* = 276	*n* = 43	*p* Value
Age		55.71 (10.214)	56.04 (10.057)	57.55 (10.059)	57.046 (10.127)	0.074
Gender(m/f)		568/533	99/90	150/126	23/20	0.916
Duration of diabetes(in years)		3.75 (5.11)	9.364 (6.20)	7.756 (6.14)	11.205 (6.166)	0.00
Hba1c		7.90 (2.43)	9.475 (2.22)	8.424 (2.21)	9.323 (2.229)	0.00
BMI range(mean)		14–44 (25.89)	15.41–51.95 (24.19)	14.82–39.73 (25.03)	16.65–33.75 (24.455)	0.001
**Lipid Profile**						
Serum Cholesterol mmol/L		4.80 (1.05)	4.862 (1.16)	4.919 (0.965)	5.11 (1.021)	0.094
Serum TGL cholesterol mmol/L		1.75 (1.151)	1.748 (1.006)	1.719 (1.12)	1.877 (0.918)	0.271
Serum HDL cholesterol mmol/L		0.99 (0.254)	1.046 (0.274)	1.066 (0.262)	1.054 (0.202)	0.00

Total number of patients: *n* = 1561. Abbreviations: HbA1c—glycated haemoglobin; BMI—body mass index; TG—triglycerides; HDL—high density lipoprotein.

**Table 5:** AUC for the model trained and tested on different subsets (rows: training set, columns: evaluation set).

	val	tsA	tsDR−	tsDR+
**trA**	0.8013 ± 0.0257	0.7097 ± 0.0031	0.7105 ± 0.0032	0.8673 ± 0.0088
**trDR−**	0.7842 ± 0.0334	0.6944 ± 0.0139	0.6941 ± 0.0145	0.8733± 0.0504
**trDR+**	0.6878 ± 0.0945	0.5993 ± 0.0410	0.5981 ± 0.0485	0.6805 ± 0.0587

Abbreviations: Rows (training sets): trA: trained on all images; trDR−: trained on images of patients without DR; trDR+: trained on images of patients with DR. Columns (test sets): val: validation set (same distribution as the corresponding training set); tsA: all test images; tsDR−: patients without DR; tsDR+: test images containing only images of patients with DR.

## Data Availability

Not applicable.
